# Biomarkers and Endothelial Damage in Obesity: An Insight into the Pharmacological Modulation

**DOI:** 10.3390/ijms27083694

**Published:** 2026-04-21

**Authors:** Arturo Yonatan Bojórquez-González, Eduardo Gómez-Sánchez, Daniel Osmar Suarez-Rico, Alberto Beltrán-Ramírez, Luis Ricardo Balleza-Alejandri, Luis Daniel López-Murillo, Ernesto Javier Ramírez-Lizardo, Jesús Jonathan García-Galindo

**Affiliations:** 1 Doctorado en Farmacología, Centro Universitario de Ciencias de la Salud, Universidad de Guadalajara, Guadalajara 44340, Mexico; arturo.bojorquez@academicos.udg.mx (A.Y.B.-G.); luis.balleza3286@alumnos.udg.mx (L.R.B.-A.); 2 Departamento de Fisiología, Centro Universitario de Ciencias de la Salud, Universidad de Guadalajara, Guadalajara 44340, Mexico; eduardo.gsanchez@academicos.udg.mx; 3 Instituto de Terapéutica Experimental y Clínica, Centro Universitario de Ciencias de la Salud, Universidad de Guadalajara, Guadalajara 44340, Mexico; daniel.suarez@academicos.udg.mx (D.O.S.-R.); alberto.beltran@academicos.udg.mx (A.B.-R.); luis.lopez0239@academicos.udg.mx (L.D.L.-M.); ernesto.rlizardo@academicos.udg.mx (E.J.R.-L.)

**Keywords:** atherosclerosis, endothelial dysfunction, Netrin-1, GLP-1 agonists, SGLT2 inhibitors, ICAM-1, endocan

## Abstract

Obesity drives chronic low-grade inflammation and endothelial dysfunction, key contributors to subclinical atherosclerosis. This review focuses on the netrin 1/UNC5B axis and its role in promoting macrophage retention within adipose tissue and atherosclerotic plaques, thereby perpetuating local inflammation and vascular injury. Complementary inflammatory markers—including IL 6, hsCRP, and IL 15—are discussed as indicators of systemic inflammatory burden, whereas endocan and ICAM 1 are briefly addressed as markers of endothelial activation. Among emerging pharmacological strategies, glucagon-like peptide-1 receptor agonists (GLP 1RAs) and sodium-glucose cotransporter 2 inhibitors (SGLT2is) show the most consistent evidence for improving these biomarkers and reducing endothelial damage, with GLP 1RAs demonstrating direct effects on carotid intima–media thickness. Integrating biomarker profiling with obesity phenotypes may improve early risk stratification and support more precise management of subclinical atherosclerosis.

## 1. Introduction

Obesity is a chronic, complex, and relapsing disease, recognized as such by the World Health Organization and numerous medical societies [1]. Its magnitude is pandemic and affects all ages. A recent global analysis from the Global Burden of Disease (GBD) Study 2021 estimated that, approximately 1.00 billion adult men and 1.11 billion adult women were living with overweight or obesity, with a prevalence of 45.1% [2]. In parallel, that same year, 173.7 million children and adolescents had overweight or obesity, and the prevalence of obesity in this group tripled since 1990 [3]. Today, obesity is defined as a disorder characterized by excessive or abnormal distribution of adipose tissue that impairs health [1].

Its pathophysiology is multifactorial, involving complex interactions among genetics, neuroendocrine regulation of appetite and satiety—where interindividual variability in satiation (calories consumed until fullness) has been shown to have a strong genetic component and to predict response to anti-obesity medications—environmental factors, and behavioral aspects [4,5,6].

Excess adipose tissue is not merely an inert deposit but rather an endocrinologically active tissue that promotes a state of chronic low-grade inflammation, characterized by elevated biomarkers such as high-sensitivity C-reactive protein (hsCRP), interleukin-6 (IL-6), interleukin-15 (IL-15), netrin-1 (Ntn1), among other inflammatory markers. As a result, these can lead to insulin resistance and metabolic dysfunction, progressively affecting multiple organs such as the vascular endothelium and participating in the progression of atheromatous disease [5,7].

Atheromatous disease is a chronic inflammatory disease that progresses over years, and in many cases is only detected in patients who develop symptoms of cardiovascular disease such as myocardial ischemia. One of the main mechanisms by which obesity fosters the onset and progression of atheromatous plaque is the recruitment and immunomodulation of M1 phenotype macrophages; the retention of these macrophages, resulting from the activation of Unc-5 netrin Receptor B (UNC5B) in the tissue and promoted by proinflammatory molecules, leads to endothelial dysfunction and atherogenesis [8,9].

It has been observed that Ntn1 increases in early stages of diabetes and in obesity, maintaining a direct relationship with the inflammatory state and glucose spikes. These findings reinforce the idea that Ntn1 actively participates in the triad of inflammation, insulin resistance, and cardiovascular complications, ceasing to be a simple marker and becoming a central player in metabolic damage [10,11]. In contrast, adiponectin, an adipokine with anti-inflammatory and insulin-sensitizing effects, shows reduced serum levels in clinical obesity with insulin resistance, establishing an opposite profile to that of Ntn1 [11].

This Ntn1/adiponectin axis represents a key immunometabolic marker in the transition toward metabolic dysfunction [11]. It is important to highlight the dual role of Ntn1 in cardiovascular disease: while at the systemic (plasma) level it may have an atheroprotective effect by inhibiting leukocyte adhesion to the endothelium, its local production by macrophages within adipose tissue and the atherosclerotic plaque promotes the retention of these macrophages, preventing their emigration and thus perpetuating local inflammation, plaque progression, and plaque instability [12,13]. Indeed, in patients with coronary artery disease, intracellular Ntn1 levels in macrophages positively correlate with macrophage accumulation in the atheromatous plaque assessed in vivo [12].

Visceral obesity, assessed using measures such as waist circumference (WC) or waist-to-height ratio (WHtR), is a particularly strong marker of cardiometabolic dysfunction and adverse clinical outcomes [4,14,15]. In fact, in conditions such as heart failure, the so-called “obesity paradox” (better prognosis with higher BMI) appears to be attenuated or to disappear when indices that better reflect central adiposity, such as WHtR, are used and adjusted for key prognostic factors [14].

Phenotypic classification based on metabolic and weight status has allowed the identification of subgroups with differential cardiovascular risk. Among these, metabolically healthy obesity (MHO) and metabolically unhealthy obesity (MUO) have been most extensively characterized [15,16]. A recent prospective cohort study demonstrates that these phenotypes present differential risks for heart failure (HF) subtypes: MUO is associated with an increased risk of both heart failure with preserved ejection fraction (HFpEF) and reduced ejection fraction (HFrEF), whereas MHO is mainly associated with an increased risk of HFpEF but not HFrEF [16]. This finding underscores that, even in the absence of overt dysmetabolism, excess adiposity—possibly through inflammatory mechanisms and abnormal fat distribution—can impair cardiovascular function, particularly diastolic function [16]. Accordingly, this review focuses on the MHO and MUO phenotypes when discussing biomarker profiles and their association with subclinical atherosclerosis. Other obesity phenotypes, such as sarcopenic or dynapenic obesity, are not covered in depth; these represent important areas for future research given their distinct pathophysiological and prognostic implications.

A recent consensus proposes a fundamental clinical distinction to guide management, complementing and contextualizing these metabolic phenotypes [1]:

1. Preclinical Obesity: Presence of excess adiposity (confirmed by BMI and another measure such as waist circumference or WHtR) [1] without evidence of related organic dysfunction. It represents a high-risk state and could include individuals with the MHO phenotype, although they may already present subclinical elevations of inflammatory biomarkers such as hsCRP, IL-6, or Ntn1 [2,10,11,12].

2. Clinical Obesity: Presence of excess adiposity together with attributable dysfunction of one or more organs or systems (e.g., metabolic—such as type 2 diabetes or insulin resistance (HOMA-IR > 2.5)—cardiovascular—atherosclerosis, hypertension—respiratory, musculoskeletal) or limitations in activities of daily living [1]. This criterion identifies manifest disease and aligns predominantly with the MUO phenotype, where chronic inflammation and proinflammatory biomarkers such as Ntn1 are usually significantly elevated (at the tissue level and, in some contexts, serum), while adiponectin is reduced, configuring an environment conducive to endothelial dysfunction and the progression of atherosclerotic disease [10,11,12].

The prognostic utility of this distinction has been empirically validated. A prospective cohort study that applied these definitions found that, compared with preclinical obesity, clinical obesity was associated with a 41% excess risk in cardiovascular disease mortality, together with objective evidence of adverse cardiac remodeling and elevation of myocardial damage biomarkers [17]. This approach shifts management from simple weight reduction toward recovery of body function, leaving behind the obsession with mere weight loss [1,18]. It thus prioritizes therapeutic interventions (pharmacological, surgical) for clinical obesity, while emphasizing prevention and health counseling in the preclinical phase [1]. Adopting this framework is essential to advance toward precision medicine in obesity, overcoming stigma and optimizing the allocation of health resources [1,19], especially given the projections indicating a continuous and substantial increase in its prevalence and disease burden globally for all ages [2,3].

Furthermore, the specific association between netrin-1 levels, central adiposity, and subclinical manifestations of atherosclerosis in patients with obesity, stratified according to their clinical phenotype (preclinical vs. clinical), has not been fully elucidated. This review aims to: (1) synthesize current evidence on inflammatory and endothelial biomarkers associated with subclinical atherosclerosis in obesity, with emphasis on netrin-1/UNC5B, IL-15, IL-6, hsCRP, endocan, and ICAM-1; (2) discuss the mechanistic role of these biomarkers in endothelial dysfunction and carotid plaque progression; and (3) evaluate emerging pharmacological strategies that modulate these pathways, particularly in the context of clinical versus preclinical obesity phenotypes.

## 2. Inflammatory and Metabolic Biomarkers Involved in Endothelial Damage in Atherosclerosis

### 2.1. Metabolic and Immune Modulators

Interleukin-15 (IL-15) is a cytokine with relevant modulatory functions in immune cell homeostasis and activation, including populations involved in vascular inflammation. In the context of atherosclerosis, IL-15 has been implicated in the regulation of processes related to inflammatory responses within the arterial wall, particularly those involving monocytes/macrophages and chemokine-mediated signaling associated with atherogenesis. However, the literature presents inconsistent findings regarding whether its net effect is predominantly pro-atherogenic or whether, under certain conditions, it may contribute to aspects of tissue integrity, warranting cautious interpretation of clinical evidence and a clear distinction between association and causality [20].

Carotid intima–media thickness (IMT) measured by ultrasound is a widely used, noninvasive marker for identifying early or subclinical atherosclerosis. In a retrospective cohort of patients with obesity and NAFLD/steatosis, serum IL-15 concentrations were higher than in young healthy controls and were significantly associated with IMT. In multivariable analyses incorporating visceral adiposity and traditional cardiovascular risk factors (age, sex, smoking, lipid profile, and insulin resistance), only age and IL-15 remained independent predictors of IMT. These findings suggest that IL-15 may reflect an immune-inflammatory component relevant to early vascular progression in this metabolic phenotype [21].

### 2.2. Systemic Inflammatory Biomarkers

Interleukin-6 (IL-6) and high-sensitivity C-reactive protein (hsCRP) are classical and widely validated biomarkers of systemic inflammation, playing a central role in the pathophysiology of atherosclerosis and cardiovascular risk stratification. Unlike mediators more directly involved in intraplaque cellular dynamics, such as netrin-1 or IL-15, IL-6 and hsCRP reflect the overall intensity of the inflammatory state that chronically acts on the vascular endothelium [22].

IL-6 is a pleiotropic cytokine produced by multiple cell types, including macrophages, adipocytes, and endothelial cells, whose expression increases in chronic inflammatory conditions such as obesity and early atherosclerosis. Systemically, IL-6 induces hepatic synthesis of acute-phase proteins, most notably CRP. Measurement of hsCRP enables detection of subtle yet clinically meaningful increases in inflammation, even in apparently healthy individuals, consolidating its role as a marker of subclinical atherosclerosis and an independent predictor of cardiovascular events [23].

From a vascular perspective, elevated IL-6 and hsCRP levels are associated with persistent endothelial activation, increased oxidative stress, and reduced nitric oxide bioavailability—processes that precede and promote intima–media thickening and silent plaque progression. In obesity, inflamed adipose tissue represents a major source of IL-6, contributing to chronically elevated hsCRP levels and amplifying endothelial injury. Although IL-6 and hsCRP do not provide direct information on specific mechanisms such as macrophage retention or UNC5b-mediated signaling, their value lies in integrating multiple local inflammatory signals into a measurable systemic phenotype closely linked to atherosclerosis progression. In this sense, IL-6 and hsCRP complement biomarkers such as netrin-1 and IL-15, enabling a more comprehensive view of the inflammation–endothelium axis in subclinical atherosclerosis [22,24].

### 2.3. Emerging Biomarkers

Netrin-1 is a laminin-related protein, originally described as an axonal guidance cue and regulator of neuronal migration, encoded by the NTN1 gene on chromosome 17. Within the cardiovascular system, netrin-1 has emerged as a key modulator of vascular inflammation, exerting effects that are highly dependent on the cellular source and the receptor involved [25].

Under acute conditions, endothelial-derived netrin-1 can attenuate leukocyte adhesion and migration, thereby contributing to the limitation of inflammatory responses. In contrast, within the chronic microenvironment of the atherosclerotic plaque, foam cell macrophages express and secrete netrin-1 together with its receptor UNC5 homolog B (UNC5b), establishing an autocrine/paracrine circuit that inhibits macrophage egress and perpetuates inflammation [23].

Lipid loading and exposure to oxLDL induce the expression of NTN1 and UNC5b in macrophages through CD36-dependent signaling and NF-κB activation. Functionally, netrin-1/UNC5b signaling blocks chemotaxis directed by CCL2 and by CCR7 ligands (CCL19/CCL21), interfering with cytoskeletal reorganization and Rac1 activation. This chemostatic state explains the retention of macrophages within the atheromatous plaque, a central feature of chronic atherosclerotic inflammation and persistent endothelial injury [22].

In humans, higher intracellular levels of netrin-1 and UNC5b in macrophages are associated with greater macrophage accumulation in the endothelial region and, consequently, with more complex plaque morphological features detectable by imaging techniques, even at subclinical stages. These findings directly link netrin-1/UNC5b signaling to endothelial dysfunction and early disease progression [26].

Adipose tissue represents a relevant source of netrin-1, where it promotes macrophage retention, chronic inflammation, and insulin resistance. This metabolic–inflammatory axis converges on the endothelium, amplifying systemic inflammatory stress and accelerating atherogenesis [27].

Recent preclinical evidence demonstrates that selective deletion of UNC5b in macrophages reduces plaque burden and complexity, improves efferocytosis, and promotes a pro-resolving environment, even after cholesterol normalization. These findings position UNC5b as an attractive therapeutic target to modulate atherosclerotic inflammation without interfering with the protective functions of endothelial-derived netrin-1 [25].

### 2.4. Clinical Integration and Limitations

Subclinical atherosclerosis represents an early stage of the disease characterized by persistent vascular inflammation, incipient endothelial dysfunction, and progressive macrophage accumulation within the intima, even in the absence of clinical events. In this context, netrin-1 has emerged as a relevant mediator linking metabolic and inflammatory signals to cellular dynamics within the atheromatous plaque. Experimental studies have demonstrated that foam cell macrophages present in early lesions express netrin-1 and its receptor UNC5b, particularly in response to oxLDL and hypoxic conditions typical of the atherosclerotic microenvironment. These stimuli activate NF-κB–dependent pathways, promoting transcription of NTN1 and UNC5b. Functionally, activation of the netrin-1/UNC5b axis inhibits directed macrophage migration toward chemotactic signals associated with plaque egress, establishing a state of cellular retention that favors chronic inflammation from early stages [13,25,28].

In humans, the relevance of this mechanism has been reinforced by studies analyzing monocyte-derived macrophages and plaque characteristics assessed by intracoronary imaging. Patients with coronary artery disease exhibit reduced plasma levels of netrin-1 but increased intracellular expression of netrin-1 and UNC5b in macrophages, a finding associated with greater macrophage accumulation within the plaque and with more complex plaque morphological features. Importantly, these characteristics are detectable even at subclinical stages, prior to overt plaque instability [29].

From a pathophysiological perspective, the persistence of retained macrophages within the intima sustains local production of cytokines, reactive species, and proteases, thereby amplifying endothelial dysfunction and promoting the silent progression of atherosclerosis. Collectively, the available evidence suggests that netrin-1, through UNC5b, is not only associated with the presence of subclinical atherosclerosis but also actively contributes to its early maintenance and progression [24].

Obesity is recognized as a major determinant of cardiovascular risk, largely due to the establishment of chronic low-grade inflammation originating in adipose tissue. In this setting, netrin-1 emerges as a key mediator linking metabolic dysfunction with vascular inflammation and endothelial injury in obese individuals. Expanded adipose tissue develops regions of hypoxia, which activate inflammatory signaling pathways dependent on NF-κB and HIF-1α. Under these conditions, resident and recruited macrophages within adipose tissue upregulate netrin-1 and its receptor UNC5b. Analogous to what is observed in atherosclerotic plaques, activation of the netrin-1/UNC5b axis inhibits directed macrophage migration, promoting local macrophage retention and perpetuating adipose tissue inflammation [30].

This macrophage retention directly contributes to the development of insulin resistance through sustained release of proinflammatory cytokines and mediators that interfere with insulin signaling. Systemically, the resulting metabolic inflammation increases the circulating inflammatory burden, directly affecting the vascular endothelium. Endothelial exposure to this proinflammatory environment leads to persistent activation, increased oxidative stress, and functional alterations that favor subclinical atherosclerosis [26].

Experimental and translational evidence suggests that netrin-1 acts as a mechanistic link between obesity and atherosclerotic disease. UNC5b-mediated signaling reinforces an inflammatory phenotype in both adipose tissue and the vascular wall, establishing a pathological circuit that accelerates endothelial damage progression. In this regard, selective modulation of the netrin-1/UNC5b axis may represent an attractive strategy to simultaneously attenuate metabolic and vascular inflammation associated with obesity [25].

The complementarity of these biomarkers allows capture of distinct dimensions of the atherosclerotic process, ranging from local cellular dynamics to systemic inflammation, thereby offering a more integrated view of the inflammation–endothelium axis. This approach not only enhances understanding of the mechanisms underlying subclinical atherosclerosis but also opens avenues for improved risk stratification and more precise therapeutic interventions aimed at modulating inflammation without compromising physiological resolution pathways.

## 3. Endothelial Dysfunction Biomarkers

### 3.1. Endocan

Previously known as Endothelial Cell-Specific Molecule-1 (ESM-1), endocan was first reported after cloning from a human umbilical vein endothelial cell (HUVEC) cDNA library; later it was described as a present transcription in several cellular lineages, as well as human coronary artery endothelial cells (HCAEC), human pulmonary artery endothelial cells (HPAEC) and human dermal microvascular endothelial cells (HDMEVC) [31,32]. Endocan is a polypeptide of 165 amino acids, with a 50 kDa molecular weight, plus a single dermatan sulfate (DS) chain containing 32 disaccharide repeats [33,34]. Due to its structure and functions, it can be segmented into distinct regions. Amino acids 1–110 corresponds to a cysteine-rich domain with 18 cysteine residues. Within this domain, residues 1–46 share features with the extracellular domain of the epidermal growth factor receptor (EGFR) and have been implicated in tumorigenesis, and binding β-catenin [35,36]. The remaining 55–amino acid non-cysteine segment is organized into three functional modules: an endothelial growth factor-like region, a phenylalanine-rich region, and a C-terminal region [37].

Endocan has an estimated half-life of approximately 1 h and is primarily degraded by cathepsin G [38]. Cathepsin G, released by neutrophils, hydrolyzes endocan into nonglycosylated peptide fragments—1–111, 1–115, or 1–116—that are collectively referred to as P14 endocan (~14 kDa), which are readily cleared by glomerular filtration [39,40].

Endocan transcription is modulated by multiple growth factor inputs—including VEGF-A, VEGF-C, HGF/MET, FGF-2, and EGFR. These signaling axes can converge and potentiate one another, supporting angiogenesis and tumor expansion [41].

#### Endocan in Endothelial Dysfunction

In clinical research settings, several approaches quantify NO-mediated vasodilatory capacity to identify endothelial impairment [42]. Two commonly used bedside readouts include ultrasound-based brachial artery flow-mediated dilation and finger tonometry-derived peripheral reactive hyperemia [43,44]. In this context, elevated circulating endocan has been observed in individuals with higher arterial pulse wave velocity, a finding that may reflect greater arterial stiffness [45].

The glycocalyx—formed by PGs and glycoproteins—constitutes a critical endothelial surface layer lining the vascular lumen and contributes to leukocyte–endothelium interactions, thrombosis, permeability regulation, and vascular inflammatory signaling [46]. In experimental models, endocan knockdown in IL-1β–stimulated chondrocytes markedly reduces expression of angiogenesis-related mediators, including VEGF-A, MMP-9, MMP-13, and VEGFR-2, supporting a regulatory role for endocan in angiogenic and inflammatory programs [47].

Endocan has been reported to promote secretion of proinflammatory cytokines, increase reactive oxygen species (ROS) generation enhancing oxidative stress. It may also dysregulate nitric oxide (NO) signaling by suppressing the AKT/eNOS axis while activating NF-κB/iNOS signaling, mechanisms that can contribute to barrier breakdown and vascular inflammation [48].

Glycocalyx disruption is a progressive feature of endothelial dysfunction, and circulating endocan has been proposed as a biomarker of this process, with potential utility in monitoring vascular injury and treatment response [49,50,51,52,53,54].

### 3.2. ICAM-1

First described in 1986, the intercellular adhesion molecule (ICAM) 1, is a transmembrane immunoglobulin (Ig) with an ~55 kDa peptide backbone, which is expressed in several cell types [55,56]. Multiple mice ICAM-1 splice products have been reported, including at least six membrane-associated isoforms and one soluble isoform. These variants differ in the number of Ig domains they contain, which in turn influences their capacity to bind LFA-1 [57]. In humans, ICAM-1 undergoes post- or co-translational N-linked glycosylation at eight sites, and these glycans can meaningfully affect ligand-binding properties [58]. Scott et al. detected a remaining functional high-mannose ICAM-1 in human coronary arteries, supporting the presence of hypoglycosylated ICAM-1 in vivo, enabling monocyte adhesion and downstream signaling [59]. Several studies reported that TNF-α stimulation induces transient expression of high-mannose ICAM-1 [60,61].

For many years, the prevailing interpretation or TNF-α was that NF-κB served as the dominant driver of ICAM-1 upregulation, but recent findings suggest that NF-κB appears to underlie early induction of ICAM-1 and other inflammatory genes and may contribute more substantially to maintaining a chronic inflammatory endothelial phenotype after cytokine withdrawal [62,63].

IFN-γ induces ICAM-1 through STAT1 homodimer binding to an IFN-γ response element. Similarly, IL-6-mediated induction involves binding of phosphorylated STAT3—either as a homodimer or as a STAT3/STAT1 heterodimer—to an element within the same promoter region [64]

#### 3.2.1. ICAM-1 in Endothelial Dysfunction

Atherosclerosis involves accumulation of lipids within the arterial wall, evolving into plaques that can enlarge to impair perfusion and cause ischemia, until abrupt plaque disruption precipitates an acute cardiovascular event [65].

Early plaque formation involves adhesion of monocytes and T cells to the endothelium and their subsequent transmigration, supporting a role for ICAM-1 and other adhesion molecules in disease initiation [66,67].

Within plaques, sustained ICAM-1 expression may reflect ongoing exposure to proinflammatory cytokines, although the mechanisms governing early endothelial induction remain under active investigation.

#### 3.2.2. ICAM-1 in Obesity Phenotypes

The expression of ICAM-1 varies across obesity phenotypes, reflecting differences in underlying inflammatory and metabolic status. A study by Liu et al. directly compared circulating ICAM-1 levels in adults classified as MHO, MUO, and normal weight controls [68]. Individuals with MUO exhibited significantly higher ICAM-1 concentrations than those with MHO, and both obese groups had higher levels than lean controls. Moreover, ICAM-1 correlated positively with markers of insulin resistance (HOMA IR) and high-sensitivity C-reactive protein (hsCRP), suggesting that the inflammatory burden associated with metabolic unhealthiness drives endothelial adhesion molecule upregulation [69]. In contrast, individuals with MHO, despite having elevated body mass index (BMI), showed only modest increases in ICAM-1, indicating a relatively preserved endothelial phenotype in the absence of overt metabolic dysfunction [68].

Weight loss interventions consistently reduce ICAM-1 levels, supporting the reversibility of obesity-related endothelial activation. A systematic review and meta-analysis by Seyyedi and Alizadeh found that surgically induced weight loss (primarily bariatric surgery) led to significant decreases in circulating ICAM-1, along with other adhesion molecules such as VCAM 1 and E selectin [70]. More recently, Andersson et al. reported that bariatric surgery in patients with obesity and type 2 diabetes reduced ICAM-1 concentrations at 12 months post surgery, and these reductions were associated with improvements in glycemic control and the glycocalyx biomarker syndecan 1 [71]. In non surgical settings, lifestyle interventions that achieve sustained weight loss have also been shown to lower ICAM-1 levels, particularly in individuals with metabolically unhealthy obesity [69,72]. Collectively, these findings indicate that ICAM-1 is a dynamic marker responsive to metabolic improvement and weight reduction, and that its elevation is more pronounced in MUO than in MHO, reinforcing the role of metabolic health in modulating endothelial dysfunction in obesity.

### 3.3. Endocan and ICAM-1 in Obesity

Multiple studies have evaluated these two biomarkers in obesity and their relevance to obesity-related vascular pathology. For ICAM-1, the available evidence consistently supports its utility as a strong indicator of endothelial dysfunction across the obesity continuum: elevations are reported across age groups and tend to improve following weight-loss interventions [69,70,71,72]. By contrast, findings regarding endocan are more context-dependent. Endocan may be particularly informative as an early marker in pediatric obesity or in selected obesity-related complications (e.g., neuropathy), yet its value as a broadly reliable biomarker in obese adults is variable—several studies report no difference, or even reduced concentrations, relative to non-obese controls [68,73].

From a mechanistic standpoint, endocan may function upstream by influencing the expression of adhesion molecules (including ICAM-1), especially under inflammatory or hypoxic conditions that are common in severe obesity or in comorbid states such as obstructive sleep apnea [74,75]. At the same time, discrepancies in circulating endocan levels across studies are plausibly driven by differences in population characteristics (age, sex, ethnicity), obesity phenotypes (metabolically healthy vs metabolically unhealthy), burden of comorbid disease (e.g., diabetes or metabolic syndrome), and methodological heterogeneity in study design and measurement [76].

## 4. New Pharmacological Interventions Interacting with Biomarkers Associated with Carotid Intima–Media Thickness

The development of pharmacological therapies targeting the pathophysiological mechanisms of subclinical atherosclerosis in obesity has experienced significant advances. These interventions act on multiple molecular pathways involved in endothelial damage, vascular inflammation, and atherosclerotic plaque progression, demonstrating interactions with key biomarkers and effects on carotid IMT.

### 4.1. Preclinical Evidence

#### Soluble Guanylate Cyclase (sGC) Agonists

sGC agonists act on the nitric oxide–cyclic guanosine monophosphate (NO-cGMP) pathway. sGC stimulators (riociguat, vericiguat) sensitize the enzyme to endogenous NO, increasing intracellular cGMP, inducing vasodilation and inhibiting vascular smooth muscle cell proliferation [77].

**Figure 1:**
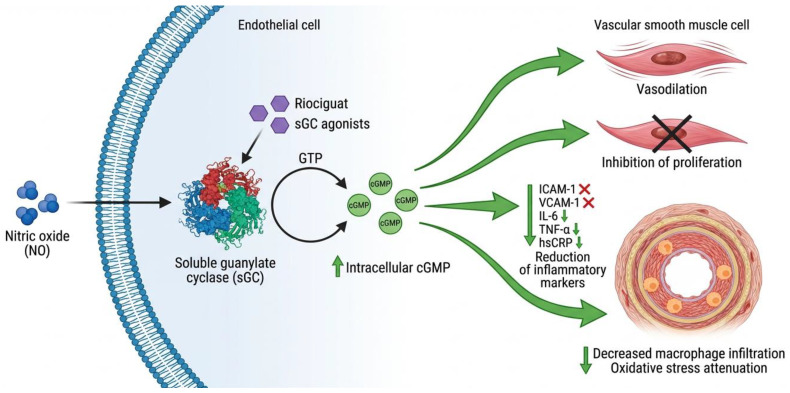
Cellular signaling mechanism of soluble guanylate cyclase (sGC) agonists in endothelial cells. Nitric oxide (NO) enters the endothelial cell and activates the soluble guanylate cyclase (sGC) enzyme. sGC agonists (riociguat and vericiguat) sensitize the enzyme to endogenous NO, enhancing the conversion of GTP (guanosine triphosphate) to cGMP (cyclic guanosine monophosphate). Increased intracellular cGMP levels trigger a cascade of therapeutic effects: vasodilation through vascular smooth muscle relaxation, inhibition of smooth muscle cell proliferation, reduction in inflammatory markers (ICAM-1, VCAM-1, IL-6, TNF-α, hsCRP), decreased macrophage infiltration in the arterial wall, and oxidative stress attenuation. This mechanism is relevant for the treatment of endothelial dysfunction in obesity and subclinical atherosclerosis, although clinical evidence on effects on carotid intima–media thickness (cIMT) in patients with obesity is still limited. sGC, soluble guanylate cyclase; NO, nitric oxide; cGMP, cyclic guanosine monophosphate; ICAM-1, intercellular adhesion molecule-1; VCAM-1, vascular cell adhesion molecule-1; IL-6, interleukin-6; TNF-α, tumor necrosis factor-alpha; hsCRP, high-sensitivity C-reactive protein.

Preclinical studies demonstrated that sGC-cGMP activation reduces ICAM-1, VCAM-1, IL-6, TNF-α, and hsCRP, associating with decreased macrophage infiltration in the arterial wall [78]. Oxidative stress is also attenuated [79]. Clinical evidence on effects on carotid IMT in obesity is limited (Figure 1).

### 4.2. Clinical Evidence

SGLT2i were developed to reduce renal glucose reabsorption in diabetes. However, large clinical trials in patients with diabetes and/or heart failure have revealed consistent cardiovascular benefits (reduced hospitalizations for heart failure, kidney disease, etc.), even in patients without diabetes [80,81]. These benefits suggest “pleiotropic” actions on non-glucoregulatory cells. A key hypothesis is their direct effect on the endothelium: preclinical studies show that SGLT2i enhance endothelium-dependent vasodilation, increase eNOS-mediated NO production, and mitigate oxidative stress and vascular inflammation, increase endothelial viability, improve mitochondrial homeostasis and angiogenesis, and reduce the expression of proinflammatory markers [81,82,83,84]. Clinically, improvements in endothelial function biomarkers (e.g., FMD, cfPWV) have been observed with these drugs [85,86].

**Figure 2:**
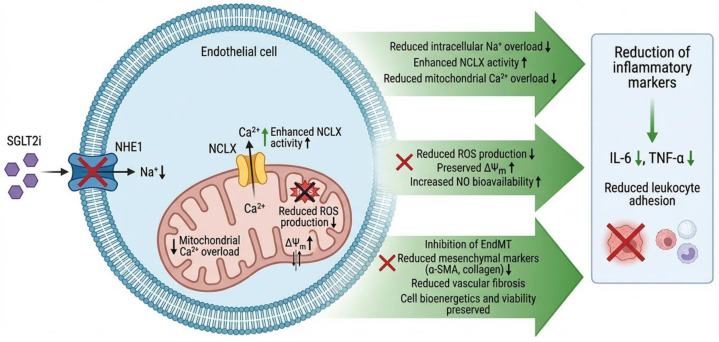
Mitochondrial protection mechanism mediated by SGLT2i (sodium-glucose link transporter 2 inhibitors) in endothelial cells. SGLT2i inhibits the NHE1 (sodium-proton cotransporter 1) transporter (blue) in endothelial cells, which would reduce intracellular Na^+^ overload, altered ion gradient favors the activity of the mitochondrial NCLX (Na^+^/Ca^2+^ exchanger) (yellow) and, consequently, mitochondrial oxidative stress. This results in lower ROS (reactive oxygen species) production, preserving NO (nitric oxide) bioavailability. sGC, soluble guanylate cyclase; NO, nitric oxide; cGMP, cyclic guanosine monophosphate; ICAM-1, intercellular adhesion molecule-1; VCAM-1, vascular cell adhesion molecule-1; IL-6, interleukin-6; TNF-α, tumor necrosis factor-alpha; hsCRP, high-sensitivity C-reactive protein.

Proposed molecular mechanisms include the indirect inhibition of the sodium-proton cotransporter 1 (NHE1) in endothelial cells (Figure 2), resulting in a decrease in cytosolic sodium concentration (Na^+^) [87]. This altered ion gradient favors the activity of the mitochondrial Na^+^/Ca^2+^ exchanger (NCLX), facilitating the extrusion of calcium (Ca^2+^) from the mitochondrial matrix [88]. Preventing mitochondrial calcium overload reduces the production of reactive oxygen species (ROS) and allows for the restoration of the mitochondrial membrane potential (ΔYm), thus preserving cell bioenergetics and viability [88]. In fact, recent studies show that SGLT2i decrease endothelial–mesenchymal transition (EndMT), a pathological process in which endothelial cells acquire pro-fibrotic fibroblastic feature, as demonstrated by in vitro studies showing that dapagliflozin and empagliflozin inhibit TGF-β-induced EndMT, reducing the expression of mesenchymal markers (α-SMA, collagen) in HUVECs. Furthermore, they observed that both drugs attenuate cardiac fibroblast activation (reduced proliferation and migration). Taken together, these data suggest that SGLT2i modulate multiple endothelial pathways: on the one hand, they suppress the inflammatory response (lowering IL-6, TNF-α, and leukocyte adhesion); on the other, they inhibit EndMT and vascular fibrosis [89,90].

It is important to emphasize that, while SGLT2i indirectly improve endothelial function through mild ketosis and natriuresis [91], there is evidence of glucose-independent effects. In cell and animal models, SGLT2i protect the endothelium even without changes in glucose levels, indicating a direct action. This set of biological mechanisms supports their current use in diabetes and heart failure and opens the door to research into additional indications (e.g., atherosclerosis prevention) via the endothelial mechanism [92].

### 4.3. Emerging Therapies

#### 4.3.1. Aldosterone Synthase Inhibitors

**Figure 3:**
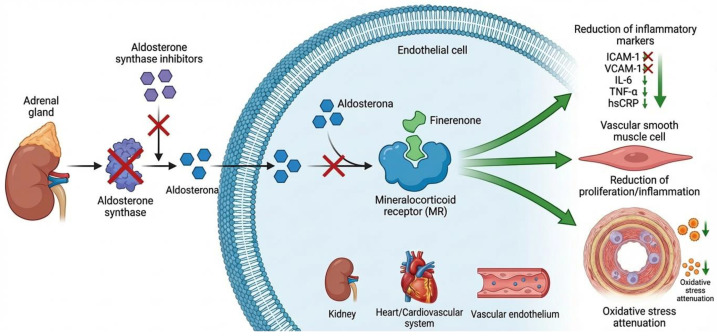
Cellular signaling mechanism of aldosterone synthase inhibitors and finerenone (mineralocorticoid receptor antagonist) in endothelial cells. Aldosterone synthase inhibitors block aldosterone production in the adrenal gland. Aldosterone normally activates the mineralocorticoid receptor (MR) in endothelial, inflammatory, and vascular smooth muscle cells, promoting proinflammatory and profibrotic effects. Finerenone, a non-steroidal MR antagonist, directly blocks MR activation in target cells, preventing the deleterious effects of aldosterone. Increased intracellular levels of finerenone bound to MR trigger a cascade of therapeutic effects: reduction in inflammatory markers (ICAM-1, VCAM-1, IL-6, TNF-α, hsCRP), decreased oxidative stress, reduction in endothelial dysfunction, and decreased vascular inflammation and fibrosis in the vascular endothelium, kidney, and cardiovascular system. This dual mechanism is relevant for the treatment of chronic kidney disease, type 2 diabetes, and obesity. Limited evidence exists on the potential for beneficial effects on carotid intima–media thickness (cIMT) in patients with obesity. sGC, soluble guanylate cyclase; NO, nitric oxide; cGMP, cyclic guanosine monophosphate; ICAM-1, intercellular adhesion molecule-1; VCAM-1, vascular cell adhesion molecule-1; IL-6, interleukin-6; TNF-α, tumor necrosis factor-alpha; hsCRP, high-sensitivity C-reactive protein.

Non-steroidal mineralocorticoid receptor antagonists, such as finerenone, significantly reduce hsCRP and IL-6 in patients with chronic kidney disease and type 2 diabetes [93]. Specific evidence on carotid IMT in obesity is scarce (Figure 3).

#### 4.3.2. GLP-1 Receptor Agonists

**Figure 4:**
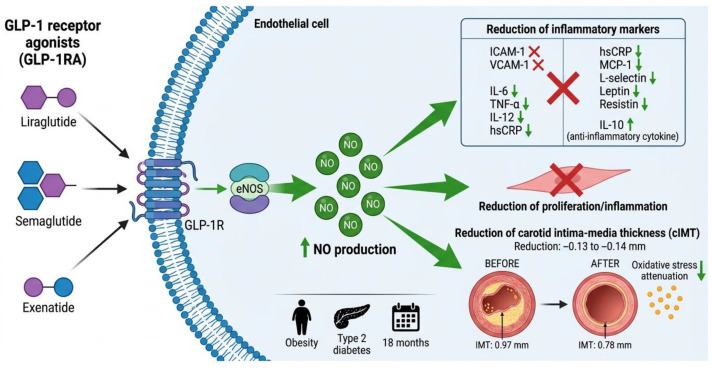
Cellular signaling mechanism of GLP-1 receptor agonists (GLP-1RA) in endothelial cells. GLP-1RA (liraglutide, semaglutide, exenatide) activate the GLP-1 receptor (GLP-1R) on the cell membrane, triggering activation of endothelial nitric oxide synthase (eNOS) and increased nitric oxide (NO) production. This mechanism generates pleiotropic effects: (1) Anti-inflammatory effects: significant reduction in multiple inflammatory markers (ICAM-1, VCAM-1, IL-6, TNF-α, IL-12, hsCRP, MCP-1, L-selectin, leptin, resistin), while increasing IL-10 (anti-inflammatory cytokine); (2) Vascular effects: improved endothelial function, reduced vascular smooth muscle proliferation and inflammation; (3) Reduction in carotid intima–media thickness (cIMT): clinical studies demonstrated that liraglutide reduces cIMT from 0.97 mm to 0.78 mm in 18 months, and exenatide produces reductions of −0.13 to −0.14 mm; (4) Attenuation of oxidative stress in the arterial wall. These effects are relevant for the treatment of obesity, type 2 diabetes, and subclinical atherosclerosis. Clinical evidence in obese patients with 18-month follow-up supports these cardiovascular benefits. sGC, soluble guanylate cyclase; NO, nitric oxide; cGMP, cyclic guanosine monophosphate; ICAM-1, intercellular adhesion molecule-1; VCAM-1, vascular cell adhesion molecule-1; IL-6, interleukin-6; TNF-α, tumor necrosis factor-alpha; hsCRP, high-sensitivity C-reactive protein.

GLP-1 receptor agonists (GLP-1RA) have emerged as a promising intervention for subclinical atherosclerosis in obesity, exerting pleiotropic effects on endothelial function and vascular inflammation (Figure 4).

GLP-1RA improve endothelial function through increased endothelial nitric oxide and eNOS activation [94]. Luna-Marco et al. (2023) reported that treatment decreased ICAM-1, VCAM-1, IL-6, TNF-α, and IL-12, while increasing IL-10 [95]. Ren et al. (2025) confirmed that GLP-1RA significantly reduce hsCRP and IL-6 [96]. Lobo et al. (2024) demonstrated that semaglutide reduces hsCRP with SMD of −0.56 (95% CI: −0.69 to −0.43) versus placebo [97]. Simental-Mendía et al. (2021) reported reduction in leptin and resistin [98]. Hachuła et al. (2024) found significant decrease in MCP-1 and L-selectin (*p* < 0.001) [99].

The most robust evidence comes from liraglutide. Nikolic et al. (2022) demonstrated over 18 months that liraglutide significantly reduced carotid IMT in obese patients (from 0.97 ± 0.17 mm to 0.78 ± 0.22 mm, *p* < 0.0001) and non-obese patients with type 2 diabetes [100]. Vergès et al. (2022) confirmed IMT reduction from 0.97 mm to 0.78 mm after 18 months [94].

A network meta-analysis by Lv et al. (2024) showed that exenatide significantly slowed IMT progression (MD = −0.13, 95% CI: −0.25 to −0.01 vs. placebo) [101]. Yanai et al. (2023) reported that exenatide reduced IMT by −0.14 mm (95% CI: −0.25 to −0.02; *p* = 0.016) [102]. Sakiz et al. (2022) found improvements in the atherogenic plasma index and uric acid with exenatide [103].

#### 4.3.3. Tirzepatide (Dual GIP/GLP-1 Agonist)

Tirzepatide, a dual GIP/GLP-1 agonist, demonstrated superior effects on weight reduction and cardiometabolic parameters. A post hoc analysis showed that tirzepatide 10 and 15 mg significantly reduced YKL-40, ICAM-1, and leptin versus placebo and dulaglutide, with rapid reductions at 4 weeks [104].

**Figure 5:**
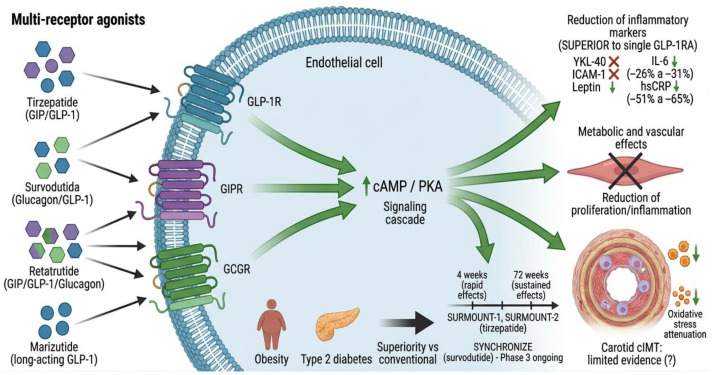
Cellular signaling mechanism of multi-receptor agonists in endothelial cells. Multi-receptor agonists (tirzepatide, survodutide, retatrutide, marizutide) activate multiple receptors on the cell membrane: tirzepatide activates GLP-1R and GIPR (dual glucagon/GLP-1 agonist); retatrutide activates GLP-1R, GIPR, and GCGR (triple agonist); marizutide is a long-acting GLP-1 agonist. Multi-receptor activation triggers intracellular signaling cascades (cAMP/PKA) that generate therapeutic effects superior to single GLP-1RA: superior reduction in inflammatory markers (YKL-40, ICAM-1, leptin, IL-6 −26% to −31%, hsCRP −51% to −65%), greater weight loss, cardiometabolic improvement, reduced vascular smooth muscle proliferation and inflammation, and oxidative stress attenuation. Effects are rapid (4 weeks) and sustained (72 weeks). SURMOUNT-1 and SURMOUNT-2 studies with tirzepatide; SYNCHRONIZE studies with survodutide in Phase 3. Limited evidence on effects on carotid intima–media thickness (cIMT). These multi-receptor agonists are relevant for the treatment of obesity and type 2 diabetes with superior cardiovascular benefits.

Sattar et al. (2024) reported in SURMOUNT-1 and SURMOUNT-2 that tirzepatide significantly reduced IL-6 (−26% to −31%; −16% to −23%) and hsCRP (−51% to −65%; −55% to −56%) at 72 weeks [105]. These anti-inflammatory effects were primarily associated with weight reduction (Figure 5).

#### 4.3.4. Survodutide (Dual Glucagon/GLP-1 Agonist)

Survodutide, a dual glucagon/GLP-1 agonist, is being evaluated in phase 3 SYNCHRONIZE studies in patients with obesity [106,107,108]. Wang et al. (2026) proposed a conceptual framework to operationalize disease modification in obesity trials based on SYNCHRONIZE-1 [109]. Results on biomarkers and carotid IMT are pending publication (Figure 5).

#### 4.3.5. Retatrutide, Marizutide, and Triple Agonists

Retatrutide, a triple GIP/GLP-1/glucagon agonist, demonstrated significant effects on weight reduction. Milan et al. (2024) reported marked decrease in body weight and improvements in cardiometabolic biomarkers, including hsCRP [110]. Marizutide represents a long-acting GLP-1 agonist. Zhou et al. (2025) developed a triple GLP-1/GIP/glucagon agonist for obesity and atherosclerosis [111]. Evidence on carotid IMT for these agents is limited (Figure 5).

#### 4.3.6. Amylin Agonists

Amylin agonists regulate food intake and gastric emptying, demonstrating beneficial effects on weight reduction. Evidence on subclinical atherosclerosis biomarkers and carotid IMT in obesity is very limited.

### 4.4. Conclusions and Future Perspectives

In obesity-related cardiovascular damage lies a persistent, low-grade inflammation that slowly affects endothelial function and sets the stage for subclinical atherosclerosis. As we have seen throughout this review, adipose tissue actively secretes inflammatory mediators that reach the vascular endothelium and disrupt its balance [5,7,9].

This process involves two interconnected layers of inflammation. On one side, systemic markers such as IL 6 and hsCRP capture the overall inflammatory load that chronically stresses the vessel wall. Their elevation reliably tracks with endothelial activation, reduced nitric oxide availability, and thickening of the carotid intima–media, making them well validated sentinels of cardiovascular risk [22,23,24]. On the other side, local players operate within the tissue microenvironment. Netrin 1 and its receptor UNC5B are produced by macrophages inside the atheromatous plaque, where they act like an anchor, preventing these immune cells from leaving [12,13,25,30]. This retention of macrophages fuels ongoing inflammation and directly links the inflamed adipose tissue to progressive plaque buildup. ICAM 1, expressed on activated endothelial cells, further amplifies the process by helping monocytes stick to and migrate through the endothelium—a crucial early step in atherogenesis [66,67].

What ties these observations together is the concept of macrophage retention as a common thread between obesity and atherosclerosis. When netrin 1/UNC5B signaling keeps macrophages trapped in adipose tissue and in the arterial wall, it creates a self-sustaining loop of local inflammation that systemic markers alone cannot fully capture.

From a therapeutic standpoint, this mechanistic understanding opens doors for intervention. GLP 1 receptor agonists and SGLT2 inhibitors have emerged as promising tools, not only because they improve metabolic parameters but also because they consistently reduce the inflammatory biomarkers discussed here and, in the case of GLP 1RAs, have been shown to regress carotid IMT [80,81,82,83,84,85,86,94,95,96,97,98,99,100,101,102]. Still, these drugs should be seen as part of a broader strategy—one that aims to restore endothelial health and interrupt the inflammatory cascade at its roots.

Looking ahead, combining detailed biomarker profiles—covering both systemic and local mediators—with a clearer characterization of obesity phenotypes (such as metabolically healthy versus unhealthy obesity) could help us move toward earlier, more personalized interventions. The path forward will require long-term studies that track how changes in these biomarkers relate to actual cardiovascular outcomes, as well as dedicated trials to see whether newer multi-receptor agonists can match or exceed the vascular benefits already observed with GLP 1RAs and SGLT2is.

## 5. Limitations

Several limitations should be considered when interpreting the evidence synthesized in this review.

Heterogeneity in study populations and obesity definitions. Across the studies discussed, there is considerable variation in how obesity is defined (BMI, waist circumference, waist-to-height ratio) and in the classification of metabolic phenotypes (e.g., MHO vs. MUO) [1,15]. Differences in inclusion criteria, age, sex, ethnicity, and comorbidity burden (e.g., type 2 diabetes, hypertension) further contribute to heterogeneity, complicating direct comparisons and meta-analytic synthesis.

Lack of standardized biomarker cutoffs. For most biomarkers reviewed—including netrin-1, IL-15, endocan, and ICAM-1—there are no universally accepted reference ranges or clinically validated thresholds that define “elevated” risk in the context of obesity. This limits the ability to translate biomarker findings into clinical decision-making and underscores the need for large, well-phenotyped cohort studies to establish normative values.

Limited data on multi-receptor agonists and carotid IMT. While GLP-1 receptor agonists have robust evidence supporting reductions in carotid intima–media thickness [100,101,102], data for newer multi-receptor agonists (e.g., tirzepatide, survodutide, retatrutide) are still emerging. Current evidence is largely derived from biomarker substudies (e.g., IL-6, hsCRP) rather than direct imaging endpoints [105,110]. Whether these agents favorably modify carotid IMT or other structural markers of subclinical atherosclerosis remains to be established in dedicated trials.

Predominance of cross-sectional rather than longitudinal data. Many of the associations between biomarkers and subclinical atherosclerosis reported in the literature are derived from cross-sectional studies [10,11,21]. Consequently, causal inference is limited, and the temporal relationship between biomarker elevation and the onset or progression of endothelial damage cannot be firmly established. Few longitudinal studies have examined whether changes in biomarkers over time correlate with changes in carotid IMT or cardiovascular events in obesity populations.

Collectively, these limitations point out the need for future research to adopt standardized phenotyping, longitudinal designs, and outcome-focused trials to refine the role of biomarkers and targeted therapies in the management of obesity-related subclinical atherosclerosis.

## Data Availability

No new data were created or analyzed in this study. Data sharing is not applicable to this article.

## Abbreviations

The following abbreviations are used in this manuscript:

ACVR2A	Activin A receptor type IIA
BMI	Body mass index
cAMP	Cyclic adenosine monophosphate
CCL2	C-C motif chemokine ligand 2
CCL19	C-C motif chemokine ligand 19
CCL21	C-C motif chemokine ligand 21
cGMP	Cyclic guanosine monophosphate
cIMT	Carotid intima–media thickness
CRP	C-reactive protein
EGFR	Epidermal growth factor receptor
eNOS	Endothelial nitric oxide synthase
ESM-1	Endothelial cell-specific molecule-1
FDA	Food and Drug Administration
FGF-2	Fibroblast growth factor 2
FMD	Flow-mediated dilation
GBD	Global Burden of Disease
GDF	Growth differentiation factor
GIP	Glucose-dependent insulinotropic polypeptide
GLP-1	Glucagon-like peptide-1
GLP-1RA	Glucagon-like peptide-1 receptor agonist
HCAEC	Human coronary artery endothelial cells
HDL-C	High-density lipoprotein cholesterol
HF	Heart failure
HFpEF	Heart failure with preserved ejection fraction
HFrEF	Heart failure with reduced ejection fraction
HGF/MET	Hepatocyte growth factor/mesenchymal–epithelial transition factor
HIF-1α	Hypoxia-inducible factor 1-alpha
HOMA-IR	Homeostatic model assessment of insulin resistance
HPAEC	Human pulmonary artery endothelial cells
hsCRP	High-sensitivity C-reactive protein
HUVEC	Human umbilical vein endothelial cells
ICAM-1	Intercellular adhesion molecule-1
IL-1β	Interleukin-1 beta
IL-6	Interleukin-6
IL-10	Interleukin-10
IL-12	Interleukin-12
IL-15	Interleukin-15
IMT	Intima–media thickness
LDL	Low-density lipoprotein
LFA-1	Lymphocyte function-associated antigen 1
MCP-1	Monocyte chemoattractant protein-1
MHO	Metabolically healthy obesity
MUO	Metabolically unhealthy obesity
NAFLD	Nonalcoholic fatty liver disease
NF-κB	Nuclear factor kappa-light-chain-enhancer of activated B cells
NHE1	Sodium-hydrogen exchanger 1
NO	Nitric oxide
NOX4	NADPH oxidase 4
Ntn1	Netrin-1
oxLDL	Oxidized low-density lipoprotein
PI3K/Akt	Phosphoinositide 3-kinase/protein kinase B
PKA	Protein kinase A
Rac1	Rac family small GTPase 1
ROS	Reactive oxygen species
SGLT2	Sodium-glucose cotransporter 2
SGLT2i	Sodium-glucose cotransporter 2 inhibitor
SMAD	Suppressor of mothers against decapentaplegic
SNAC	Sodium N-(8-[2-hydroxybenzoyl]amino) caprylate
STAT1	Signal transducer and activator of transcription 1
STAT3	Signal transducer and activator of transcription 3
T2DM	Type 2 diabetes mellitus
TGF-β	Transforming growth factor-beta
TNF-α	Tumor necrosis factor-alpha
UNC5B	Unc-5 netrin receptor B
VCAM-1	Vascular cell adhesion molecule-1
VEGF	Vascular endothelial growth factor
VEGFR-2	Vascular endothelial growth factor receptor 2
WC	Waist circumference
WHtR	Waist-to-height ratio
YKL-40	Chitinase-3-like protein 1
